# Real-world evidence of *RET* fusion prevalence and testing in patients with metastatic non-small cell lung cancer

**DOI:** 10.1016/j.tranon.2026.102854

**Published:** 2026-06-13

**Authors:** B.N. Cajiao Garcia, V.D. de Jager, C.C.H.J. Epskamp-Kuijpers, A.J. van der Wekken, S.M. Willems, L.C. van Kempen, E. Schuuring

**Affiliations:** a Department of Pathology and Medical biology, University of Groningen, University Medical Center Groningen, Groningen, the Netherlands; b Foundation Palga, Houten, the Netherlands; c Department of Pulmonary Disease and Tuberculosis, University of Groningen, University Medical Center Groningen, Groningen, the Netherlands; d Department of Pathology, University of Antwerp, Antwerp University Hospital, Edegem, Belgium

**Keywords:** Non-small cell lung cancer, *RET* fusion, Predictive biomarker testing, Selpercatinib, Pralsetinib

## Abstract

**Objectives:**

Rearranged during transfection (*RET*) fusion is a driver aberration in non-small cell lung cancer (NSCLC). Approval of *RET* inhibitors selpercatinib and pralsetinib prompts adequate molecular testing for *RET* fusions in NSCLC. The aim of this study was to determine changes in the detection of *RET* fusions and the effect of various testing strategies in stage IV NSCLC patients using real-world data.

**Materials and methods:**

Patients diagnosed with metastatic non-squamous NSCLC in 2017 and 2019 were selected from Netherlands Cancer Registry (NCR) database and linked to their pathology reports form the Dutch nationwide pathology databank (Palga). Based on these data, *RET* fusion testing rates, prevalence and laboratory variation were evaluated.

**Results:**

A total of 3651 patients and 3934 patients were included in 2017 and 2019, respectively. The prevalence of *RET* fusion in non-squamous NSCLC was 1.3% and 0.7%, in 2017 and 2019, respectively. The overall *RET* fusion testing rate increased from 22.6% (2017) to 31.8% (2019). Between 2017 and 2019, a shift from FISH (91% to 47%) to RNA-based testing (6% to 46%) for the detection of *RET* fusions was observed.

**Conclusion:**

The prevalence calculated from real-world data in the Netherlands with nation-wide coverage was 1.3% in 2017 and 0.7% in 2019. The decrease in detected *RET* fusions between 2017 and 2019 was associated with decreased use of FISH testing The prevalence of *RET* fusions in non-squamous NSCLC published in the literature (1–2%) is therefore likely overestimated.

## Highlights

• *RET* fusion prevalence in NSCLC may be overestimated at 1–2%.
• RNA-based NGS is more accurate than FISH for detection of *RET* fusions.
• The testing rate for *RET* fusions is expected to further increase in the Netherlands.
• The prevalence of *RET* fusions is expected to be lower than 1%.

## Introduction

Lung cancer causes most cancer-related deaths worldwide, with 1.8 million deaths reported in 2020 [1]. The most common type of lung cancer, non-small cell lung cancer (NSCLC), accounts for 85% of all cases [2]. Approximately 70% of NSCLC can be characterized as non-squamous. Often diagnosed at an advanced stage of disease, the five-year overall survival rate of metastatic (stage IV) non-squamous NSCLC is 10–20% in the Netherlands [1,3,4]. Non-squamous NSCLC may harbor specific molecular driver aberrations for which effective targeted therapies are available [5,6]. These targetable molecular aberrations include mutations in epidermal growth factor receptor (*EGFR*), Kristen rat sarcoma viral oncogene homolog (*KRAS*) G12C, v-Raf murine sarcoma viral oncogene homolog B1 (*BRAF*) V600, ErbB-2 receptor tyrosine kinase 2 (*ERBB2*), and mesenchymal-epithelial transition (*MET*) exon 14 skipping mutations and fusion of anaplastic lymphoma kinase (*ALK*), ROS proto-oncogene 1 (*ROS1*), rearranged during transfection (*RET*), and neurotrophic receptor tyrosine kinase 1/2/3 (*NTRK1*/*2*/*3*) [[7], [8], [9]].

*RET* is a proto-oncogene that encodes for a receptor tyrosine kinase involved in the activation of Mitogen-Activated Protein Kinase (MAPK), Phosphoinositide 3-kinase/AKT Serine/Threonine Kinase 1 (PI3K/AKT) and Janus kinase/signal transducer and activator of transcription (JAK-STAT) pathways [10]. In NSCLC, *RET* fusions have been described with more than forty-five different fusion partners, though most *RET* fusions constitute a fusion between *RET* and Kinesin family member 5B (*KIF5B)* [[11], [12], [13]]. The chimeric fusion protein results in ligand-independent, expression and constitutive activation of RET signaling driving cell proliferation, growth and survival [14]. *RET* fusions were first identified as oncogenic drivers in non-squamous NSCLC in 2012 with an estimated prevalence of 1% to 2% [[15], [16], [17], [18], [19], [20]]. Patients with NSCLC harboring a *RET* fusion are characterized by relatively young age (early 60’s), non-smoking history and high rates of brain metastases at diagnosis [18,21,22]. Initially, fluorescent in situ hybridization (FISH) was the commonly used method to detect genomic rearrangement of *RET* [20,37,38]. While functional *RET* fusions are relevant for treatment, not all *RET* rearrangements detected by FISH result in expression of the fusion product and downstream activation of RET-related cascades [23]. As a result, RNA-based *RET* fusion detection assays are currently recommended [20,38].

Based on the results of the LIBRETTO-001 and ARROW trials, selpercatinib and pralsetinib were approved by the European Medicine Agency (EMA) in 2021 and by the Food and Drug Administration in 2020 [24,25] for their use in patients with *RET* fusion-positive metastatic NSCLC [[24], [25], [26], [27], [28]]. As a result, the European Society for Medical Oncology (ESMO) and National Comprehensive Cancer Network (NCCN) guidelines now include recommendations for *RET* fusion testing in patients with NSCLC [29,30]. From 2015 to 2020, the Dutch NSCLC treatment guidelines advised *RET* fusion testing to be tested sequentially, if *EGFR, KRAS*, and *ALK* testing results were negative. In addition, a team of Dutch experts strongly recommended molecular testing for *RET* fusions in stage IV NSCLC to ensure inclusion of patients in clinical trials [31]. As of 2020, the Dutch NSCLC treatment guidelines include routine *RET* testing for all patients with non-squamous NSCLC [32].

Despite (inter)national guidelines recommendations for *RET* fusion testing, the overall testing rate for *RET* fusions in the period preceding the inclusions in the national guidelines was low, with 20% in Q4 of 2017 in the Netherlands [33] and 27% between 2015 and 2019 in Germany [34]. The aim of the current study was to determine *RET* fusion testing rates and results in patients with metastatic non-squamous NSCLC in the Netherlands in 2017 and 2019 using real-world data from national databases.

## Methods

### Patient selection

Data were collected from two databases with nationwide coverage: the Netherlands Cancer Registry (NCR) (managed by Netherlands Comprehensive Cancer Organisation (IKNL)) and the Dutch Nationwide Pathology Databank (Palga). The NCR, a nationwide registry of patients diagnosed with cancer in the Netherlands, was queried to select all adult patients diagnosed with metastatic non-squamous NSCLC in 2017 or 2019. Clinical data from the NCR were then linked to the respective pathology reports of the Palga databank by a trusted third party (ZorgTTP, Houten, the Netherlands). Data on (molecular) testing and testing results were manually extracted from the pathology reports. Patients were excluded if they were diagnosed outside the study period, had an autopsy report only or were diagnosed with squamous NSCLC. This study was approved by the Palga privacy and scientific committee (application number: LZV2018-​199, LZV2021_101) and the ethical committee of the NCR-governing body (application numbers: K18.311, K18311_3). Data were processed in accordance with the General Data Protection and Regulation (GDPR).

### Data extraction from pathology reports

Palga reports were analyzed manually to collect data on the following variables: type of tumor specimen that was tested (histological, cytological or both), histopathological diagnosis and *RET* testing data, including whether *RET* testing was performed (≤3 months after diagnosis), testing technique, result of the test and timing of *RET* testing with respect to *EGFR* testing (in parallel or sequential). Testing technique was subdivided into DNA fluorescent in situ hybridization (FISH), RNA-based testing or both.

### Statistical analysis

*RET* fusion testing rates were divided into three subgroups: (1) all patients tested for *RET*, (2) patients that tested wildtype (wt) for *RET* and wt for other tested biomarkers (*EGFR, KRAS, BRAF* V600*, ERBB2, ALK, ROS1*) and (3) patients tested for *RET* who also carry a driver gene mutation (*EGFR, KRAS, BRAF* V600*, ERBB2, ALK, ROS1*). The frequencies of the testing methods (FISH or RNA-based NGS) that were used were calculated for each study year. Prevalence of *RET* fusions was calculated for the entire Dutch NSCLC population by extrapolating observed prevalence of *RET* fusions in tested patients to those that did not carry a driver gene mutation and were not tested for *RET* fusions. This was performed according to a cross-tabulation and formula adapted from measure of association [35] (supplement I). Difference between the *RET* tested population and stage IV NSCLC Dutch patients was tested using Chi-square statistical test [36]. Interlaboratory variations were calculated for individual laboratories with a 99% confidence interval. Statistical analysis was performed using IBM SPSS Statistics for Windows, version 28.0.1.0 (SPSS Inc., Chicago, IL, USA) and figures were made using Microsoft Excel (version 2403).

## Results

### Patient characteristics

A total of 3746 (for 2017) and 4048 patients (for 2019) with metastatic non-squamous NSCLC were retrieved from the National Cancer Registry (NCR) (supplement II). After the exclusion criteria were applied, 3651 and respectively 3934 patients were included. Patient characteristics of the entire cohort, as well as *RET* fusion-tested patients are presented in Table 1. Significant differences (*p* < 0.05) were observed between all patients diagnosed with stage IV NSCLC and patients tested for the characteristics: sex, type of laboratory and tissue type. Only diagnosis did not differ significantly. An increase in the population tested for *RET* from 22.6% to 31.8% was observed from in 2017 to 2019.

**Table 1:** Characteristics of patients diagnosed with metastatic non-squamous NSCLC in 2017 and 2019.

	2017		2019	
	All patients diagnosed with stage IV NSCLC (n = 3651)	Patients tested for *RET* (n = 825)	All patients diagnosed with stage IV NSCLC (n = 3934)	Patients tested for *RET* (n = 1251)
**Mean age in years** (±SD)	68.0 (±10.2)	67.0 (±11.0)	68.0 (±10.3)	67.0 (±10.4)
**Sex**				
Male	53.4% (n = 1951)	59.0% (n = 487)	52.7% (n = 2075)	57.5% (n = 719)
Female	46.6% (n = 1700)	41.0% (n = 338)	47.3% (n = 1859)	42.5% (n = 532)
**Diagnosis**				
Adenocarcinoma	86.7% (n = 3166)	86.2% (n = 711)	87.1% (n = 3426)	87.0% (n = 1088)
NSCLC – NOS	12.7% (n = 464)	13.3% (n = 110)	12.0% (n = 473)	12.2% (n = 153)
Adenosquamous carcinoma	0.5% (n = 18)	0.4% (n = 3)	0.6% (n = 23)	0.6% (n = 7)
NSCLC-neuroendocrine	0.1% (n = 3)	0.1% (n = 1)	0.3% (n = 12)	0.2% (n = 3)
**Type of laboratory**				
Academic	10.3% (n = 376)	16.5% (n = 136)	8.4% (n = 339)	12.3% (n = 154)
Non-academic	89.7% (n = 3275)	83.5% (n = 689)	91.4% (n = 3595)	87.7% (n = 1097)
**Tissue type**				
Histology (+/- cytology)	67.4% (n = 2459)	76.0% (n = 627)	67.1% (n = 2639)	71.9% (n = 899)
Cytology	32.6% (n = 1192)	24.0% (n = 198)	32.9% (n = 1295)	28.1% (n = 352)

SD, standard deviation; NSCLC – NOS, non-small cell lung cancer – not otherwise specified.

**2017.** sex: *p* < 0.05, χ2 (1, 4476) = 8.5, diagnosis: *p* > 0.05, χ2 (3, 4476) = 1.0, type of laboratory: *p* < 0.05, χ2 (1, 4476) = 25.4, tissue type: *p* < 0.05, χ2 (1, 4476) = 23.5.

**2019.** sex: *p* < 0.05, χ2 (1, 5185) = 8.5, diagnosis: *p* > 0.05, χ2 (3, 5185) = 1.0, type of laboratory: *p* < 0.05, χ2 (1, 5185) = 15.0, tissue type: *p* < 0.05, χ2 (1, 5185) = 10.0.

### *RET* fusion testing rates

The overall testing rates for *RET* fusions were 23% (825/3651) in 2017 and 32% (1251/3934) in 2019. Testing rates in patients without a reported activating mutation in *EGFR, KRAS, BRAF, ERBB2, ALK* or *ROS1* were 73.9% in 2017 and 78.2% in 2019. In contrast, only 8.8% of patients diagnosed in 2017 with a mutation in one of the other predictive markers were tested for *RET* fusions, and this increased to 16.8% in 2019 (supplement II).

### Order of *RET* fusion testing

**Fig. 1:**
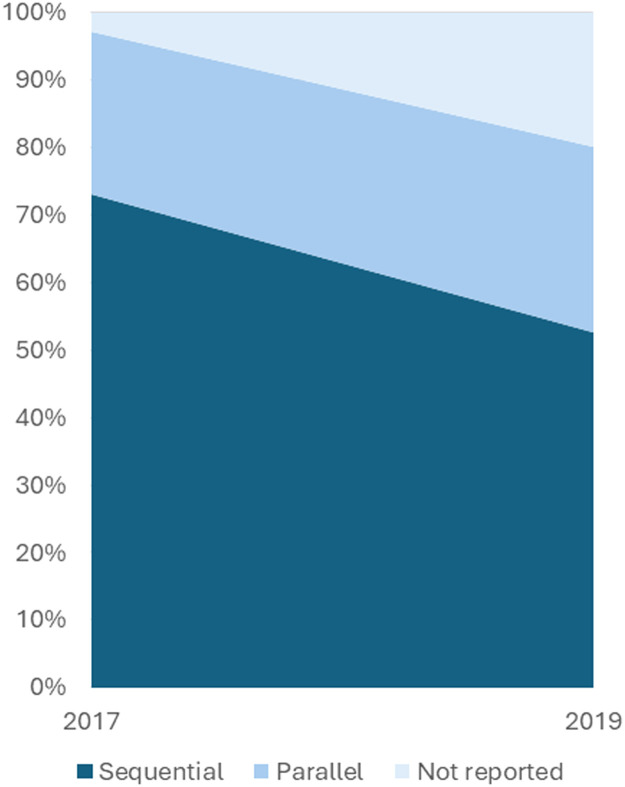
Order of *RET* fusion testing in patients with metastatic non-squamous NSCLC in 2017 and 2019. Sequential, tumor specimens tested for other predictive biomarkers (at least *EGFR*) prior to *RET* fusion testing; parallel, *RET* and other predictive markers tested in simultaneously; not reported, samples tested for *RET* fusion but test status or date of test request of other predictive markers is unknown.

In 2017, *RET* was tested in a sequential manner for 73.1% of cases (i.e., *RET* fusion testing was performed only if *EGFR, KRAS*, and *ALK* testing results were negative) (Fig. 1). In 2019, sequential testing of *RET* decreased to 52.6%, while parallel testing increased to 27.6%. For the remaining cases, testing order was unknown (2.9% in 2017 and 19.8% in 2019).

### *RET* fusion testing assays

**Fig. 2:**
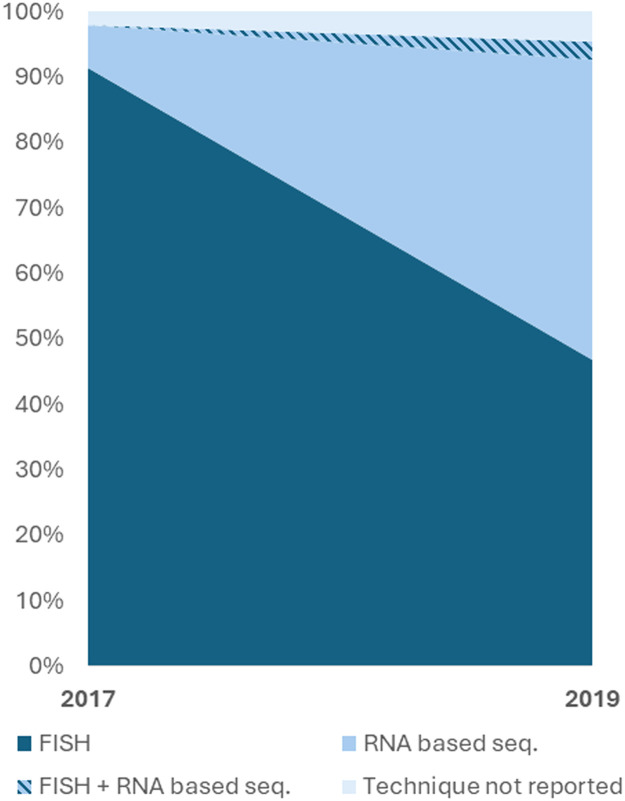
Techniques used for *RET* fusion testing in patients with metastatic non-squamous NSCLC in 2017 and 2019. Technique not reported comprises cases for which test technique was not reported in the pathology report. FISH, fluorescent in situ hybridization.

In 2017, *RET* FISH was performed in 91.3% of *RET*-tested patients, while only 6.4% of patients were tested using an RNA-based test. In 2.1% of the patients, test type was either not reported or not retrievable from the available reports. In 2019, the use of FISH decreased to 47.0%, whilst RNA-based testing increased to 46.0%. In 4.6% of the patients, the assay was not reported or not retrievable from the available reports. The remaining 2.7% of patients were tested with both FISH and an RNA-based assay (Fig. 2). In 2017, three patients with *RET* fusion-positive FISH test results had a co-occurring (driver) mutation (*ALK* fusion, *EGFR* exon 19 deletion*,* and *PIK3CA* mutation) (Supplement IIIA and IIIB).

### Prevalence of *RET* fusions

The prevalence of *RET* fusions in the *RET*-tested population was 2.3% in 2017 (19/825) and 1.2% in 2019 (15/1251). Estimated prevalences of *RET* fusions in the entire patient cohorts were calculated by extrapolating for the patients who did not carry a mutation in another predictive biomarker and who were not tested for *RET* fusions. The calculated prevalences of *RET* fusions were 1.3% and 0.7% in 2017 and 2019, respectively (Supplement I).

### Interlaboratory variation for *RET* fusion testing

**Fig. 3:**
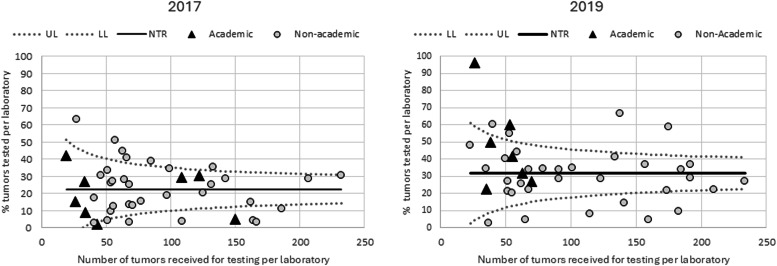
Funnel plots demonstrating interlaboratory variation in *RET* testing with 99% confidence intervals in (A) 2017 and (B) 2019. CI, Confidence interval; NTR, National testing rate.

Testing rates of individual laboratories were evaluated for the 2017 and 2019 cohorts. Thirteen laboratories scored outside the 99% confidence interval (CI), of which seven below the lower limit of the CI (Figs. 3A and B). In 2019, twelve laboratories were outside the 99% CI, of whom six were below the lower limit of the CI.

## Discussion

Nationwide real-world data from the NCR and the Palga were used to establish molecular predictive testing rates, assess test techniques and determine the prevalences of *RET* fusions in patients with metastatic (stage IV) non-squamous NSCLC diagnosed in the Netherlands in 2017 and 2019. The prevalence of *RET* fusions calculated from these data was 1.3% in 2017 and 0.7% in 2019. This decrease was associated with the reduced use of FISH testing, which has been described to yield higher false positive rates compared to RNA-based fusion testing [23]. Our analysis demonstrates that the previously reported prevalence of *RET* fusions in NSCLC (1–2%) [[15], [16], [17], [18], [19], [20]] is likely overestimated.

In both 2017 and 2019, FISH was commonly used for the detection of *RET* fusions (91.3% and 47.0%, respectively). However, back in 2019, it was not yet known that FISH was unspecific for detection of clinically relevant *RET* fusions [23], thereby necessitating orthogonal testing of positive cases [23,37]. The assessment of FISH patterns associated with true rearrangements in tissue sections is challenging and requires specific expertise [38]. In addition, FISH assays do not provide information regarding gene fusion partners nor on whether these genomic *RET* rearrangements are actually expressed [[39], [40], [41]]. As a result, the use of *RET* FISH may lead to reporting of non-functional *RET* fusions [23]. Reporting of false-positive *RET* fusion testing results inevitably leads to an overestimation of *RET* fusion prevalence, which is reflected by the findings of our study. In 2017, more *RET* fusion-positive cases were detected compared to 2019 (19 compared to 15 cases), while *RET* fusion testing rates were higher in 2019. RNA-based NGS also enables simultaneous analysis of other relevant markers (e.g., *ALK, ROS1, MET-skipping* and *NTRK1*/*2*/*3*)*,* reducing turnaround time and using tissue more efficiently than sequential application of single gene tests [42,43]. The ESMO 2021 guideline [20] and the Dutch 2023 cieBOD-guideline [44] recommend RNA-based NGS-based testing for the detection of *RET* fusions for the reason described above.

Previous studies have described *RET* fusion prevalences at 1–2% [[15], [16], [17], [18], [19], [20],^45^] in patients with NSCLC. This prevalence is usually reported from the observed *RET* fusion positive patients within the total cohort not taking into account case selection that might affect the prevalence [33,45]. In some studies, it was estimated that in patients with no mutations or fusions in other predictive markers, *RET* fusion prevalence was 5% [46,47]. However, the latter studies concerned Asian populations, which have been described with different prevalences for other driver mutations (e.g., *EGFR*) compared to European populations, and thus direct comparison is not appropriate. In our study, the prevalences of *RET* fusions in the entire patient cohorts were estimated by correcting for case selection. *RET* fusion prevalence in the *RET*-tested population was extrapolated to patients that were not tested for *RET* fusions and had no other reported driver mutations (supplement I). These calculations resulted in estimated population-wide prevalences of 1.3% for the 2017 cohort (vs. 2.3% in the *RET*-tested population) and 0.7% for the 2019 cohort (vs. 1.2% in the *RET*-tested population).

The 2015 Dutch NSCLC guidelines advised *RET* fusion testing in patients with advanced-stage NSCLC, if no mutations were detected in *EGFR, KRAS* and *ALK* [32]. At the time of these guidelines, treatment of *RET* fusion-positive NSCLC was limited for inclusion in clinical trials and compassionate use programs. In total, 21 laboratories in 2017 and 20 laboratories in 2019 had a testing rate for *RET* fusion above the national testing rate (Figs. 3A and B). The patient cohort not tested for *RET* fusions is likely due to sequential rather than parallel testing, with sequential testing more commonly applied in 2017 compared to 2019. Furthermore, other reasons for not testing involves inadequate tissue quality or poor health status, the patient is unable to undergo a tissue biopsy procedure due to their physical condition. In case no (appropriate) tumor tissue is available, the Dutch committee for the Assessment of Diagnostic (cieBOD) in June 2024 recommended the use of plasma-based circulating cell-free DNA testing using similar NGS-based assays that include the testing for *RET* rearrangements [48]. By 2023 and 2022, the ESMO and NCCN guidelines also recommend *RET* fusion testing, respectively [29,30].

An analysis of *RET* testing rates reported a lower rate between 40%−50% for a Dutch cohort of NSCLC patients that tested negative for *EGFR, KRAS, BRAF, MET, ERBB2* in Q4 of 2017*.* The discrepancy in testing rates is attributed to the sample selection of the NSCLC population and the negative genes [33]. In a similar study conducted in German patients reported a testing rate similar to this study for *RET* testing, increasing from 20% to 30% between 2016–2019 [34]. The increase in testing rates for RET fusions in the Netherlands could be attributed to availability of EMA targeted therapies selpercatinib and pralsetinib already in 2015, the use of RNA-based NGS and uptake of RET testing recommendations in the national guidelines. Recently, we reported an overview of the development and application of targeted therapies and clinical-relevant predictive biomarker testing in European patients with advanced stage NSCLC in 2023 including RET-fusion testing. Using data from 11 European countries, we found that since 2023 the EMA-approved selpercatinib and pralsetinib are available and recommended in the national guidelines in all these 11 EU countries. Because these RET-directed targeted drugs were included in the recommendations only recently, also in 2023 none of the other 9 countries had information on RET-fusion testing rates or its prevalences. But interestingly, the commonly-used RET-fusion detection assays were FISH and targeted RNA-NGS in 4, and only targeted RNA-NGS in 7 EU countries. For more comprehensive real-world data on RET fusion testing over time, this information might be collected through international External Quality Assessment programs.

A limitation of this study is the retrospective method by which data were analyzed. Here we described a group of patients for whom *RET* fusions were reported based on the FISH result. However, it is not certain whether these patients do harbor a real *RET* fusion because of the high false-positive rates of *RET* FISH testing. Based on these findings and the period the study was conducted, a prevalence lower than the one observed in 2019 is expected in the present. Moreover, although this study was conducted for an entire population, the size of this study was relatively small and compared data from 7 to  9 years ago, thus inclusion of more recent years is recommended to more accurately estimate actual prevalence. Moreover, the availability of drugs targeting RET in either regular care or via clinical trials (e.g. compassionate use program), as well as the reimbursement of this therapy, differs between various countries [9], making these findings specific for the Netherlands.

## Conclusions

Our study demonstrates that *RET* fusions in patients with non-squamous NSCLC may have a lower prevalence than what was previously been reported in literature. The decrease in detected *RET* fusions between 2017 and 2019 was associated with a decrease in *RET* FISH testing and increased use of RNA-based NGS assays. These real-world findings align with previous reports that have described *RET* FISH testing to yield higher false positive rates compared to RNA-based NGS. As over half of *RET* fusions in 2019 were still reported based only on FISH assay results, our estimated prevalence of 0.7% may yet be an overestimation of the actual prevalence of *RET* fusions in patients with NSCLC. Nonetheless, the analysis of more recent data is required to asses the impact of shifting testing methods on the prevalence and treatment outcomes of NSCLC patients.

## CRediT authorship contribution statement

**B.N. Cajiao Garcia:** Writing – review & editing, Writing – original draft, Visualization, Methodology, Investigation, Formal analysis, Data curation. **V.D. de Jager:** Writing – review & editing, Methodology, Data curation. **C.C.H.J. Epskamp-Kuijpers:** Writing – review & editing, Resources, Data curation. **A.J. van der Wekken:** Writing – review & editing, Supervision, Methodology, Formal analysis, Conceptualization. **S.M. Willems:** Writing – review & editing, Supervision, Methodology, Funding acquisition, Conceptualization. **L.C. van Kempen:** Writing – review & editing, Writing – original draft, Supervision, Methodology, Formal analysis, Conceptualization. **E. Schuuring:** Writing – review & editing, Writing – original draft, Visualization, Supervision, Investigation, Funding acquisition, Conceptualization.

## Declaration of competing interest

VdJ has received speaker fees received from Uitgeverij Jaap (Roche Diagnostics Netherlands B.V., Janssen-Cilag B.V.) (paid to UMCG account) and Canadian Anatomic and Molecular Pathology – Pathology Oncology Digital Series (Amgen, Bayer, Daiichi-Sankyo, AstraZeneca, Eli Lilly, Roche, Pfizer) (paid to UMCG account). AvdW has received grants or contracts from AstraZeneca, Boehringer Ingelheim, Pfizer, Roche, and Takeda, has received consulting fees from AstraZeneca, Janssen, Eli Lilly, Roche, and Takeda, has received payment or honoraria for lectures, presentations, speakers bureaus, manuscript writing or educational events from AstraZeneca, Bristol-Myers Squibb, Eli Lilly, Pfizer, and Roche, has a leadership or fiduciary role in oncology section NVALT, guideline committee NSCLC and CUP, dure geneesmiddelen committee NVALT and FMS. LvK has received institutional grants or contracts from Amgen, AstraZeneca, Bayer, Janssen-Cilag, Merck, Roche, and Servier, has received payments or honoraria for lectures, presentations, speakers bureaus, manuscript writing or educational events from AstraZeneca, Bayer, Bristol-Myers Squibb, Eli Lilly, Janssen, Novartis, Pfizer, and Roche (all to institution), has received support for attending meetings and/or travel from Roche and ThermoFisher (all to institution), has participated on a Data Safety Monitoring Board or Advisory Board of Cyclomics, Janssen-Cilag, LOGEX, Merck, Menarini, Protyon, and Roche (all payments to institution), has a leadership or fiduciary role in the EORTC Melanoma Group and the Commission Personalized Medicine – Belgium (all payments to institution), has stock or stock options in Cyclomics (personal). ES has received (unrestricted) grants from Abbott, Biocartis, AstraZeneca, Invitae/Archer, Bayer, Bio-Rad, Roche, Agena Bioscience, CC Diagnositcs, MSD/Merck, and SNN/EFRO (all paid to UMCG institution), has received advisory board (incidental) and travel expenses (honoraria/grant paid to UMCG institution) from MSD/Merck, AstraZeneca, Roche, Novartis, Bayer, BMS, Lilly, Amgen, Illumina, Agena Bioscience, CC Diagnostics, and Janssen Cilag (Johnson&Johnson), has received advisory board (incidental) (honoraria/grant paid to UMCG institution) from Astellas Pharma, GSK, Sinnovisionlab, Sysmex, and Protyon, has received payments or honoraria for lectures, presentations, speakers bureaus, manuscript writing or educational events from Bio-Rad (travel expenses and honoraria paid to UMCG institution), Seracare (honoraria paid to UMCG institution), Roche (travel expenses and honoraria paid to UMCG institution), Biocartis (travel expenses and honoraria paid to UMCG institution), Lilly (travel expenses and honoraria paid to UMCG institution), Agena Bioscience (travel expenses and honoraria paid to UMCG institution), and Illumina (honoraria paid to UMCG institution, has received support for attending meetings and/or travel from BioRad (only travel expenses), Biocartis (only travel expenses), Ageno Sciences (only travel expenses), Illumina (travel expenses), Roche/Foundation Medicine (travel/hotel/registration expenses (partially)), and QCMD (travel/hotel/registration expenses), has a leadership or fiduciary role in the Dutch Society of Pathology (board member, unpaid), European Society of Pathology (board member, unpaid), European Liquid Biopsy Society (board member, unpaid), is chairman/member of the Committee for assessment of molecular diagnostics (cieBOD, honoraria paid to UMCG institution), is committee member of the National guideline advisory committee (honoraria paid to UMCG institution), and is board member of the Committee for Clinical Essential Targets (cieKNT) (unpaid). SW has received grants or contracts from Roche, Bayer, Eli Lilly, Pfizer, AstraZeneca, Merck Sharp & Dohme, Amgen, and Novartis (unrestricted research grants), and has a leadership or fiduciary role in the strategic advisory board of Roche. The remaining authors declare no conflict of interest.

## Appendix Supplementary materials

Image, application 1
