# Supplementary material for: Real-world evidence of RET fusion prevalence and testing in patients with metastatic non-small cell lung cancer
Source: Transl Oncol. 2026 Jun 13;70:102854. doi: 10.1016/j.tranon.2026.102854 (PMC13279202; doi:10.1016/j.tranon.2026.102854)
Supplement: Supplementary file 1 [file mmc1.docx]

**Supplement I. Calculation for extrapolating the prevalence in the entire population using measures of association**^1^**.**

|  | D+ | D- | Total |  | 2017 | D+ | D- | Total |  | 2019 | D+ | D- | Total |
| --- | --- | --- | --- | --- | --- | --- | --- | --- | --- | --- | --- | --- | --- |
| E0 | A0 | B0 | N0 |  | **E0** | 19 | 806 | 825 |  | **E0** | 15 | 1236 | 1251 |
| E1 | A1 | B1 | N1 |  | **E1** | 26,4 | 1121,5 | 1148 |  | **E1** | 10,7 | 886,2 | 897 |
| E2 | A2 | B2 | N2 |  | **E2** | 0 | 1652 | 1652 |  | **E2** | 0 | 1660 | 1660 |
|  | M1 | M2 | N |  |  | 45,4 | 3579,5 | 3625 |  |  | 25,7 | 3782,2 | 3808 |
| Prevalence |  |  |  |  |  |  |  | 1.3% |  |  |  |  | 0.7% |
| Prevalence = M1/N = (A0 + A1 + A2)/ (N0 + N1 + N2)*100  A0= amount of *RET*-positive cases in the tested group (E0)  A1= predicted amount of *RET*-positive cases in the non-tested group (E1) = (A0/N0) x N1  A2= amount of *RET*-positive cases in the non-tested patients who were tested positive for other* driver genes (E2) = 0 (assumed)  B0= amount of *RET*-negative cases in the tested group (E0)  B1= predicted amount of *RET*-negative cases in non-tested group (E1) = (B0/N0) x N1  B2= amount of *RET*-negative cases in the non-tested patients who were tested positive for other* driver genes (E2) = N2 = N- (N0+N1)  N0= total amount of *RET* tested patients  N1= total amount of *RET* non-tested patients who were tested wild type for other* driver genes  N2= total amount of *RET* non-tested patients who were tested positive for other* driver genes  *Other driver genes: *EGFR, KRAS, BRAF* V600*, ERBB2, ALK, ROS1* | | | | | | | | | | | | | |

1. B. Burt Gerstman. *Epidemiology Kept Simple: An Introduction to Traditional and Modern Epidemiology, 3rd Edition*. 3rd ed. Wiley-Blackwell; 2013. Accessed January 9, 2024.

**Supplement II. Flow diagram of patients included, testing rates and *RET* fusion detected cases**

**
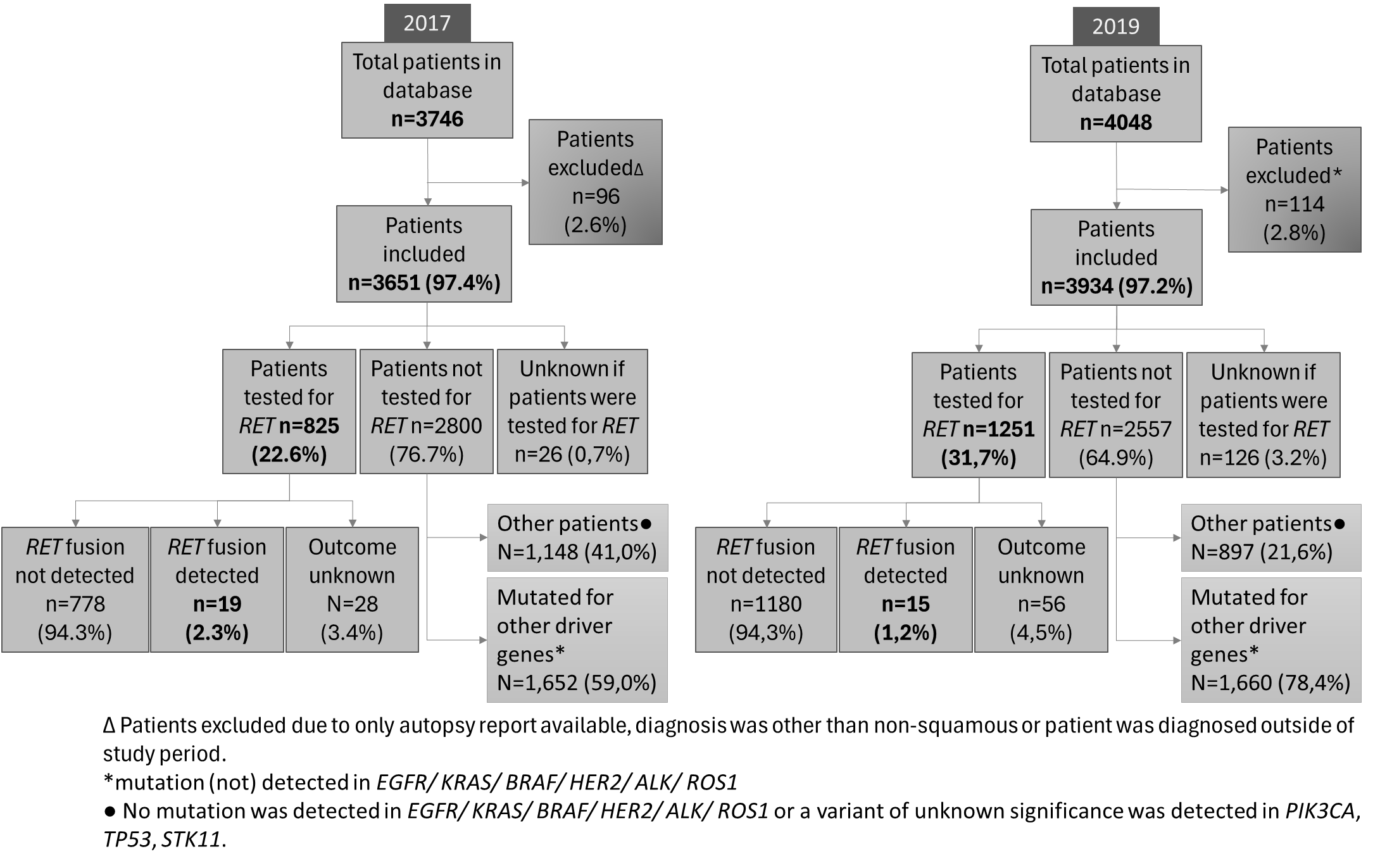
**

**Supplement IIIA**

**Table 1. *RET* rearrangements detected in mutated cohort non-squamous NSCLC patients in 2017**

| ***ID*** |  |  | ***RET fusion detection method*** | ***Breakpoint pattern (%)*** | ***KRAS/EGFR tested*** | ***Co-occurring mutation*** |
| --- | --- | --- | --- | --- | --- | --- |
| ***1*** |  |  | FISH | 78% | Yes |  |
| ***2*** |  |  | FISH | 67% | Yes |  |
| ***3*** |  |  | FISH | 46% | Yes |  |
| ***4*** |  |  | FISH | 46% | No, insufficient tissue |  |
| ***5*** |  |  | FISH | 45% | Yes |  |
| ***6*** |  |  | FISH | 27% | Yes | *EGFR* c.2235_2249del p.(E746_A750del) |
| ***7*** |  |  | FISH | 24% | Yes | *PIK3CA* (D594E) |
| ***8*** |  |  | FISH | 23% | Yes |  |
| ***9*** |  |  | FISH | 52% | Yes |  |
| ***10*** |  |  | FISH | 18% | Yes | *ALK* fusion |
| ***11*** |  |  | FISH | 50% | Yes |  |
| ***12*** |  |  | FISH | 41% | Yes |  |
| ***13*** |  |  | FISH | N.R. | Yes |  |
| ***14*** |  |  | FISH | N.R. | Yes |  |
| ***15*** |  |  | FISH | N.R. | Yes |  |
| ***16*** |  |  | Unknown | N.R. | Yes |  |
| ***17*** |  |  | FISH | N.R. | Yes |  |
| ***18*** |  |  | FISH | N.R. | Yes |  |
| ***19*** |  |  | FISH | N.R. | Yes |  |

Legenda: FISH, Fluorescent In Situ Hybridisation; N.R., not reported

**Supplement IIIB**

**Table 2. *RET* rearrangements detected in mutated cohort non-squamous NSCLC patients in 2019**

| ***ID*** |  |  | ***Technique***  ***FISH/RNA based NGS*** | ***Breakpoint pattern (%) or gene-usion*** | ***KRAS/EGFR tested*** | ***Co-occurring mutation*** |
| --- | --- | --- | --- | --- | --- | --- |
| ***1*** |  |  | FISH | N.R. | Yes |  |
| ***2*** |  |  | FISH | 26% | Yes |  |
| ***3*** |  |  | FISH | 16% | Yes |  |
| ***4*** |  |  | FISH | 18% | Yes |  |
| ***5*** |  |  | FISH | N.R. | Yes |  |
| ***6*** |  |  | FISH | N.R. | Yes |  |
| ***7*** |  |  | FISH | 76% | Yes |  |
| ***8*** |  |  | FISH | 54% | Yes |  |
| ***9*** |  |  | FISH + Nanostring | 25% | Yes |  |
| ***10*** |  |  | Nanostring | N.R. | Yes |  |
| ***11*** |  |  | NanoString | *KIF5B*::*RET* fusion | Yes |  |
| ***12*** |  |  | NGS gene fusion | *KIF5B*::*RET* fusion | Yes |  |
| ***13*** |  |  | NGS gene fusion | *KIF5B*::*RET* fusion | Yes |  |
| ***14*** |  |  | NGS gene fusion | *CCDC6*::*RET* fusion | Yes |  |
| ***15*** |  |  | NGS gene fusion | *KIF5B*::*RET* fusion | Yes |  |

Legenda: FISH, Fluorescent In Situ Hybridisation; N.R., not reported
